# Identification of a *SLC19A2* nonsense mutation in Persian families with thiamine-responsive megaloblastic anemia

**DOI:** 10.1016/j.gene.2013.02.008

**Published:** 2013-05-01

**Authors:** Aria Setoodeh, Amirreza Haghighi, Nasrollah Saleh-Gohari, Sian Ellard, Alireza Haghighi

**Affiliations:** a Growth & Development Research Centre, University of Tehran, Medical Sciences, Tehran, Iran; b The Hospital for Sick Children, University of Toronto, Toronto, Ontario, Canada; c Genetic Department, Kerman University of Medical Sciences, Kerman, Iran; d Institute of Biomedical and Clinical Science, Peninsula College of Medicine and Dentistry, University of Exeter, Exeter, UK; e Wellcome Trust Centre for Human Genetics, University of Oxford, Oxford, UK

**Keywords:** TRMA, thiamine-responsive megaloblastic anemia, PCR, polymerase chain reaction, CT scan, computerized tomography scan, MCV, mean corpuscular volume, WBC, white blood cell, SDS, standard deviation score, MPH, mid-parental height, TRMA, Thiamine-responsive megaloblastic anemia, Rogers syndrome, *SLC19A2*

## Abstract

Thiamine-responsive megaloblastic anemia (TRMA) is an autosomal recessive syndrome characterized by early-onset anemia, diabetes, and hearing loss caused by mutations in the *SLC19A2* gene. We studied the genetic cause and clinical features of this condition in patients from the Persian population. A clinical and molecular investigation was performed in four patients from three families and their healthy family members. All had the typical diagnostic criteria. The onset of hearing loss in three patients was at birth and one patient also had a stroke and seizure disorder. Thiamine treatment effectively corrected the anemia in all of our patients but did not prevent hearing loss. Diabetes was improved in one patient who presented at the age of 8 months with anemia and diabetes after 2 months of starting thiamine. The coding regions of *SLC19A2* were sequenced in all patients. The identified mutation was tested in all members of the families. Molecular analyses identified a homozygous nonsense mutation c.697C > T (p.Gln233*) as the cause of the disease in all families. This mutation was previously reported in a Turkish patient with TRMA and is likely to be a founder mutation in the Persian population.

## Highlights

► A non-sense *SLC19A2* in four patients with TRMA indicating its high frequency in Persian population. ► Most patients with this mutation had short stature. ► This study expands the knowledge about the of genotype–phenotype correlations in TRMA. ► The results have implications for genetic counseling in other affected families.

## 1 Introduction

Thiamine-responsive megaloblastic anemia (TRMA, Rogers syndrome, OMIM 249270) is a very rare autosomal recessive condition with childhood onset (Porter et al., 1969). TRMA is characterized by the clinical triad of megaloblastic anemia, sensorineural hearing loss, and diabetes mellitus (Neufeld et al., 1997). The other reported features include visual impairments, congenital heart defects, tri-lineage myelodysplasia, short stature, and stroke (Bergmann et al., 2009).

TRMA is caused by mutations in the *SLC19A2* gene (Neufeld et al., 1997). This gene is located on chromosome 1q23.3, spanning 6 exons, and encodes a high-affinity plasma membrane thiamine transporter (THTR-1) (Dutta et al., 1999). *SLC19A2* is expressed in a wide range of human tissues including the bone marrow, pancreas, brain, retina, heart, skeletal muscle, kidney, liver, lung, small intestine, colon, placenta, lymphocytes and fibroblasts (Fleming et al., 1999). Using mouse models, it has been recently demonstrated that THTR1 along with THTR2 are involved in carrier-mediated thiamine uptake by pancreatic acinar cells (Subramanian et al., 2012). In human, *SLC19A2* has been shown to encode the sole thiamine transporter in bone marrow, a subset of cochlear cells, and pancreatic beta cells (Oishi et al., 2012). *SLC19A2* mutations result in thiamine deficiency in pancreatic beta cells and other affected tissues, leading to defects in cellular metabolism, cell stress, and apoptosis (Oishi et al., 2012). High dose thiamine therapy in TRMA patients can improve the disease symptoms, correcting the anemia and reducing or cessating the need for exogenous insulin (Alzahrani et al., 2006; Porter et al., 1969). Liberman et al. (2006) demonstrated that, in mice, deletion of *SLC19A2* results in selective loss of inner hair cells of cochlea and leads to auditory neuropathy phenotype. It has been suggested that thiamine supplementation might prevent sensorineural hearing loss in children if started before two months of age (Onal et al., 2009).

We investigated the genetic basis of the diseases in four new cases of TRMA from the Persian population.

## 2 Patients and methods

### 2.1 Families and patients

We studied three Persian families with TRMA. Two families were consanguineous (first cousins) whereas the third family (with two affected siblings) was non-consanguineous. The diagnosis was made based on the presence of megaloblastic anemia, sensorineural deafness, and diabetes mellitus.

Written informed consent for clinical and molecular investigation was obtained from all members of the families or their legal guardians. The study was conducted in accordance with the Helsinki Declaration.

### 2.2 Methods

Genomic DNA was extracted from peripheral blood using QIAamp DNA blood Midi kits (Qiagen, Hilden, Germany). The exons and intron–exon boundaries of *SLC19A2* were amplified using polymerase chain reaction (PCR). Unidirectional sequencing was carried out using standard methods on an ABI 3730 sequencer (Applied Biosystems, Warrington, UK). The resulting DNA sequence was analyzed with Mutation Surveyor software (version 3.20; SoftGenetics, State College, PA) and against the reference sequence (accession number NM_006996.2).

The other family members were screened for the identified mutation.

## 3 Results

### 3.1 Clinical investigation

We identified four patients from three unrelated families. Two families were consanguineous (first cousins) but the third family, with two affected siblings (P-2 and P-3), was non-consanguineous. The clinical details are summarized in Table 1. All our patients presented with early onset anemia, hearing loss, and diabetes. Three of the patients (P2–4) presenting with anemia (with onset ages of 3 m, 1 y, and 5 y) were transfusion dependent without any precise diagnosis when their diabetes was diagnosed. The patients were born after uneventful and full-term pregnancies. The hearing problem started from infancy when their parents noted they showed no response to sounds. All parents were healthy.

On physical examination, all patients were pale and of short-stature, whereas their parents and healthy siblings had normal stature. Examinations of the heart, lungs, eyes, kidneys and liver were normal. Brainstem audiometry revealed sensorineural hearing loss in all patients.

The important laboratory findings included decreased hematocrit and hemoglobin levels, and elevated random serum glucose and fasting serum glucose levels in all probands. Serum creatinine, electrolytes, ferritin, folic acid and B12 levels, urea nitrogen, and liver enzymes were within normal ranges in all patients. The patient had normal serum amino acid levels and no aminoaciduria was detected. One of the patients, P-1, had thrombocytopenia whereas the platelet counts in other probands were within normal ranges.

The peripheral blood smear demonstrated predominance of macrocytic cells with no hemolytic findings such as fragments or spherocytes. Bone marrow aspiration was performed and histopathological study of the specimens revealed megaloblastic changes, abnormal erythropoiesis and normal cellularity, except in P-3 that showed hypocellularity.

One of the patients, P-2, had history of stroke and several seizures. The electroencephalogram, at the age of 2 years, was suggestive of focal epilepsy. His ischemic stroke at 16 months, and in presence of diabetic ketoacidosis, resulted in left hemiparesis. CT-scan and lumbar puncture were normal.

Based on clinical, including anemia and deafness, and paraclinical findings, TRMA was diagnosed and insulin therapy was started to correct the hyperglycemia. The treatment of patients with oral thiamine hydrochloride was remarkably effective in correction of anemia, and hemoglobin and hematocrit increased to normal levels within a few weeks; however no improvement in MCV values was observed (Table 1). The thrombocytopenia of P-1 was corrected. Thiamine treatment did not improve the hearing problem in any of our patients, but improved diabetes in P-1 after 2 months and therefore insulin was stopped. At the age of 24 months, HbA1C level was normal (5.9%).

### 3.2 Molecular analysis

Molecular study of the *SLC19A2* gene in the four probands revealed that all were homozygous for a nonsense mutation, c.697C > T (p.Gln233*), in the second exon. This mutation substitutes glutamine at position 233 for a stop codon, predicted to cause truncation of THTR1 and/or nonsense mediated decay of the *SLC19A2* messenger RNA.

All the obligate carrier parents were confirmed as heterozygous for the nonsense mutation.

## 4 Discussion

We identified a homozygous nonsense mutation in *SLC19A2* in a series of four TRMA patients from three unrelated Persian families. Mutation c.697C > T, alters the amino acid glutamine at residue 233 to a stop codon. This mutation was previously described in a 5 year old male patient from a non-consanguineous Turkish family (Ozdemir et al., 2002). To date, only four *SLC19A2* mutations have been identified in multiple families, of which only two (c.484C > T and c.515G > A) were present in more than one ethnicity (Bergmann et al., 2009).

Three of the 4 patients had short stature. The etiology of short stature is unclear because further workup including the evaluation of GH–IGF-I axis was not done. Thiamine therapy, however, did not improve growth of those patients.

Interestingly, hearing loss was the first recognized feature and was present at birth in three of four patients reported in this study, whereas no congenital hearing loss in TRMA has been reported in the literature, to our knowledge. Thiamine started at the age of 8 months did not prevent hearing loss in P-1 (Table 1), contrary to previous reports (Onal et al., 2009), but did improve blood sugar levels.

Significant neurocognitive deficits, stroke and seizure, were present in one of our patients, P-2, but other cases and the Turkish patient did not report similar history. His stroke, at 16 months of age, was ischemic and resulted in left hemiparesis. Stroke is a sparse feature in TRMA, reported in only a few cases, of which, only in one case genetic study was performed, revealing a nonsense mutation, G152*, as the cause of the disease (Bergmann et al., 2009). His seizures occurred in the absence of hypoglycemia, and EEG evaluations at the age of 2 years revealed focal epilepsy. Seizure has been reported only in 5 TRMA cases. To our knowledge, this is the first report of a TRMA case with coexistence of stroke and seizure disorder.

Ricketts et al. investigated 13 patients and had follow-up data for a median of 9 years (2–30 y) (Ricketts et al., 2006). They reported that almost all patients became transfusion dependent in adulthood, despite the initial improvement of their hemoglobin in answer to thiamine therapy, suggesting puberty as determinant of deteriorating the metabolic control. On contrary, all our patients maintained a good response to thiamine therapy, their hemoglobin and hematocrit levels remained within the normal ranges, and they did not become transfusion dependent in adulthood even several years after puberty (P-2 and P-3).

Early treatment with thiamine can improve diabetes in TRMA, as observed in one of our cases (P-1) and the Turkish patient. Blood sugar levels of P-1 were controlled after 2 months of treatment with thiamine, and insulin was discontinued. Treatment outcomes in this case and other Turkish patient suggest that early commencement of thiamine may improve diabetes. In late diagnosed cases, like our other 3 patients, the control of diabetes depends on the compliance of patients.

We identified a nonsense mutation, c.697C > T (p.Gln233*), in *SLC19A2* in a series of TRMA patients with congenital/early neonatal hearing loss and neonatal/early childhood diabetes and anemia. These findings confirm that TRMA should be considered in the differential diagnosis of neonatal/early childhood hearing loss, megaloblastic anemia of unknown cause, and diabetes. Childhood megaloblastic anemia without folic acid or vitamin B12 deficiency should also raise the possibility of TRMA. We suggest that early onset of thiamine can improve or prevent diabetes in patients with TRMA. The results of this study expand the current knowledge on genotype–phenotype correlations in *SLC19A2* and have applications in screening and genetic counseling.

## Conflict of interest

The authors declare that there is no conflict of interest relevant to this manuscript.

## Figures and Tables

**Table 1:** Clinical and hematological details of TRMA patients.

	P-1 (female)	P-2 (male)	P-3 (female)	P-4 (male)
Age (at last visit)	2 y	24 y	28 y	13.5 y
Consanguinity	+	−	−	+
The first complaints	Pallor, polyurea, polydipsia	Anemia	Anemia	Anemia
Onset age of anemia	8 m	3 m	1 y	5 y
Onset age of deafness	10 m	Congenital	Congenital	Congenital
Onset age of diabetes	8 m	16 m	2 y	7 y
Hemoglobin (g/dL)	F: 5.6 L (16 m later): 13.4	F: 8.1 L (23 y later): 13.8	F:5 L (27 y later): 12.7	F: 7.5 L (8.5 y later): 13.4
MCV	F: 100 L: 101.7	F: 101 L: 100	F: 106.9 L: 102	F: 102 L: 102
Reticulocyte count (%) (n ~ 0.5%–2%)	0.4	0.6	1.4	1.2
Platelet count	F: 30,000 L: 266,000 (16 m later) (thrombocytopenia)	214,000	320,000	284,000
WBC (/mm^3^)	4800	6400	6700	8600
Weight (centile)	(5th)	(5th)	(5th)	(10th)
Height (centile)	SDS: − 2.3	SDS: − 2.18	SDS: − 3.3	(10–25th) SDS: − 2.5 (corrected for the MPH)
Seizures	−	+	−	−
Stroke	−	+	−	−
Transfusion dependent before thiamine therapy	One episode of transfusion	+	+	+
Transfusion dependent after thiamine therapy	−	−	−	−
Thiamine dose (mg)	100	300	300	100
Peripheral smear	Macrocytosis, anisocytosis, target cells	Macrocytosis	Macrocytosis	Hypochromia, anisocytosis, macrocytosis
Bone marrow aspirate	Normocellular, megaloblastic change	Megaloblastic anemia	Hypocellular marrow with macrocytosis	Normocellular with macrocytosis
Serum folate concentration (ng/mL) (n > 2.5 ng/mL)	?	12.5	7.2	8.9
Vitamin B12 (pg/mL) (n ~ 140–700 pg/mL)	?	870	381	612
Ferritin (ng/mL)	?	1100 (due to repeated transfusion)	87	112
HbA1c at the last visit Nl < 6%	5.9% (2 y)	8.6% (24 y)	7% (28 y)	8.5% (13.5 y)

MCV: mean corpuscular volume, WBC: white blood cell, SDS: standard deviation score, MPH: mid-parental height, NA: not applicable, Y: years, M: month, +: presence of condition, −: absence of condition, ?: unknown, F: first visit, L: last visit.
